# Thyroid Hormone Status Regulates Skeletal Muscle Response to Chronic Motor Nerve Stimulation

**DOI:** 10.3389/fphys.2019.01363

**Published:** 2019-10-31

**Authors:** Jin Zhou, Daniel C. Parker, James P. White, Andrea Lim, Kim M. Huffman, Jia Pei Ho, Paul M. Yen, William E. Kraus

**Affiliations:** ^1^Program of Cardiovascular and Metabolic Disorders, Duke-NUS Medical School, Singapore, Singapore; ^2^Division of Geriatrics, Duke University School of Medicine, Durham, NC, United States; ^3^Claude D. Pepper Older Americans Independence Center/Center for the Study of Aging and Human Development, Duke University School of Medicine, Durham, NC, United States; ^4^Division of Hematology, Duke University School of Medicine, Durham, NC, United States; ^5^Duke Molecular Physiology Institute, Duke University School of Medicine, Durham, NC, United States; ^6^Division of Rheumatology, Duke University School of Medicine, Durham, NC, United States; ^7^Division of Cardiology, Duke University School of Medicine, Durham, NC, United States

**Keywords:** thyroid hormone, skeletal muscle, exercise, muscle fiber transition, cell signaling, chronic motor nerve stimulation, skeletal muscle metabolism

## Abstract

Although both exercise and thyroid hormone (TH) status can cause cellular and metabolic changes in skeletal muscle, the impact of TH status on exercise-associated changes is not well understood. Here, we examined the effects of TH status on muscle fiber type, cell signaling, and metabolism in a rabbit model of exercise training – chronic motor nerve stimulation (CMNS). Five rabbits were rendered hypothyroid for 7–8 weeks and three rabbits were made hyperthyroid for 2 weeks prior to CMNS of the left peroneal nerve for 10 days. We then measured markers of muscle fiber type, autophagy, and nutrient- or energy-sensing proteins, and metabolic intermediates. CMNS increased MHC-I expression in hypothyroid rabbits, whereas it was unchanged in hyperthyroid rabbits. CMNS also increased p-AMPK, p-ATGL, CPT-1α, p-Akt, GLUT4, and p-70S6K in hypothyroid rabbits. In contrast, p-AMPK and p-AKT were increased at baseline in hyperthyroid rabbits, but CMNS did not further increase them or any of the other markers. CMNS also increased TCA cycle and acylcarnitine metabolites in hypothyroid rabbits; whereas, acylcarnitines were already elevated in hyperthyroid rabbits, and were only slightly increased further by CMNS. In summary, CMNS effects on cell signaling and metabolism of skeletal muscle were more pronounced in the hypothyroid than the hyperthyroid state. Interestingly, in the hypothyroid state, CMNS caused concomitant activation of two signaling pathways that are usually reciprocally regulated – AMPK and mTOR signaling – which manifested as increased β-oxidation, MHC-I expression, and protein synthesis. Thus, our findings provide insight into the role of TH status on exercise response in muscle. Our observations suggest that TH status of patients may be an important determinant and predictor of their response to exercise training in skeletal muscle.

## Introduction

Thyroid hormone (TH) status and exercise both cause distinct cellular and metabolic changes in skeletal muscle; however, the impacts of TH status on exercise-associated changes in muscle are poorly understood. The thyroid hormones – thyroxine (T_4_) and the physiologically active form of thyroid hormone, triiodothyronine (T_3_) – have profound effects on skeletal muscle architecture, metabolism, and function (Bloise et al., 2018). Thyroid hormones bind to nuclear thyroid hormone receptors (THRs), acting as ligand-inducible transcription factors to regulate transcription of target genes. In skeletal muscle, TH directly regulates the expression of genes involved in muscle contractile protein fiber type and glucose metabolism: MHC-2a, SERCA1a, SERCA2a, GLUT-4, NADP-dependent malic enzyme (ME1), and muscle glycerol-3-phosphate dehydrogenase (mGPDH) (Desvergne et al., 1991; Weinstein et al., 1994; Dümmler et al., 1996; Ambrosio et al., 2017). The increased expression of these genes occur coincident with fiber type shifts from MHC-I to MHC-2a, and increases in both glycolytic and oxidative metabolism (Salvatore et al., 2014).

Chronic endurance aerobic exercise training also induces changes in the metabolism and fiber type of skeletal muscle, increasing MHC-I fiber type expression and fatty acid β-oxidation. Chronic motor nerve stimulation (CMNS) of the *peroneal* nerve of rabbits is a well-established animal model for exercise training (Williams et al., 1987). The *peroneal* nerve innervates the *tibialis anterior* and *extensor digitorum longus* (EDL) of the rabbit hind limb, firing at a frequency in the range of 100 Hz during normal activity. By continuously stimulating the *peroneal* nerve at a frequency of 10 Hz, a profound functional and metabolic demand is generated, leading to a shift in sarcomere fiber type from fast twitch (MHC-2) to slow twitch (MHC-I). This experimental approach offers the ability to observe the transitional phenotype between fast and slow twitch muscle over an abbreviated time span. Such a fiber type shift is accompanied by changes in calcium handling, enhanced oxidative metabolism, mitochondrial biogenesis, repression of glycolysis, changes in muscle fiber ultrastructure (increased capillary density, loss of muscle mass, and thinning of fibers), and a visible shift in apparent coloration from white to red (Eisenberg and Salmons, 1981; Williams, 1986; Williams et al., 1986; Annex et al., 1991). These changes are observed as early as 3 days and are complete by day 21 in rabbits undergoing CMNS (Eisenberg and Salmons, 1981).

Exercise training also activates cellular programs driving both anabolic (protein synthesis) and catabolic (autophagy) responses. These responses occur through activation of mechanistic target of rapamycin complex 1 (mTOR) and AMP-activated protein kinase (AMPK), respectively. Acute exercise activates AMPK through the depletion of intracellular ATP which, in turn, stimulates catabolic processes such as autophagy and fatty acid oxidation (Schwalm et al., 2015). Activation of mTOR also enhances the protein synthesis required for skeletal muscle hypertrophy (Bodine et al., 2001). However, since AMPK can inhibit mTOR, there may be competing activation of these two pathways when the drive for protein synthesis and autophagy occur concurrently during exercise training (Ferraro et al., 2014).

Despite the well-characterized effects of TH and chronic exercise on cell signaling and metabolism in skeletal muscle (Egan and Zierath, 2013; Salvatore et al., 2014; Jaspers et al., 2017), little is known about the role of TH status in mediating skeletal muscle’s structural and metabolic adaptations to exercise training. Since skeletal muscle constitutes 30–40% of lean body mass, it is a major contributor to the changes in systemic basal metabolic rate occurring during either hypo- or hyperthyroidism. In this study, we used an experimental design that allowed us the opportunity to evaluate the concomitant and competing effects of thyroid status on CMNS-mediated changes in skeletal muscle fiber type and metabolism within the same organism, which provides further insight into the molecular processes induced by exercise in skeletal muscle.

## Materials and Methods

### Thyroid Status Manipulation

**FIGURE 1:**
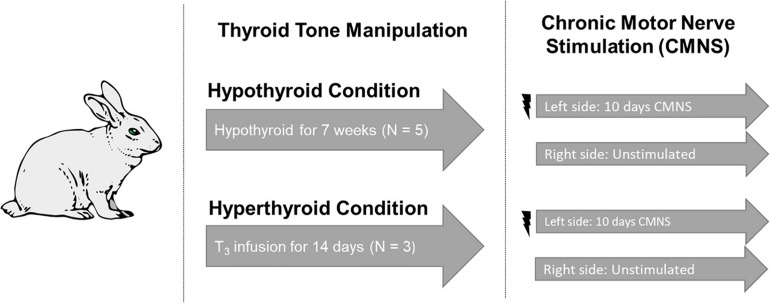
Experimental design. Five rabbits underwent total thyroidectomy and were maintained in the hypothyroid state for 7 weeks. Three rabbits received a continuous infusion of T3 for 2 weeks. Continuous stimulation with 10 Hz was applied to the peroneal nerve of the left hind limb. The unstimulated right hind from the same animal served as the paired control. Image used in Figure 1 adapted from https://pixabay.com/vectors/rabbit-bunny-hare-brown-animal-297212/.

Adult New Zealand white rabbits were studied (Figure 1). We undertook studies in rabbits because the high basal metabolic rate of rodents can mask exercise-induced changes. We chose to study the extremes of thyroid state on CMNS by creating both hyperthyroid and hypothyroid animals. Rabbits were housed within the thermo-neutral range of 15–20°C. Six rabbits underwent total thyroidectomy. In brief, rabbits were anesthetized by intramuscular injection of 0.3 ml/kg fentanyl/fluanisone (Hypnorm; Crown Chemical Co.), following an overnight fast and following premedication with 3 mg/kg atropine sulfate and 5 mg/kg diazepam. Prior to CMNS, rabbits were maintained in a hypothyroid state for 7–8 weeks. Following the same anesthetization protocol described above, six rabbits were implanted with subcutaneous osmotic pumps (ALZET^®^). A solution of thyroxine (Sigma, St. Louis, United States), was mixed in 0.025 N NaCl/150 mM NaCl solution to a concentration of 1.05 mg/mL. 2 mL of the thyroxine solution was added to the subcutaneous pumps, which had a total capacity of 2 mL. Thyroxine was delivered at an estimated rate of 50 μg/kg/day over the course the ensuing 14 days to render the animals hyperthyroid prior to CMNS. At the end of this period, plasma samples were obtained to determine thyroid status. Total and free T_3_ were quantified in sera using solid phase radioimmunoassay. Heart rate was measured using single lead electrocardiogram.

### Chronic Motor Nerve Stimulation

Our studies were performed by examining unstimulated and CMNS muscle under different thyroid states in the SAME animal. Thus, the unstimulated muscle served as the control for the stimulated muscle in each experiment. We also examined differences between hypothyroid, hyperthyroid, and euthyroid rabbits with the muscle from euthyroid rabbits serving as controls for thyroid state.

Rabbits were anesthetized by intramuscular injection of 0.3 ml/kg fentanyl/fluanisone (Hypnorm; Crown Chemical Co.), following an overnight fast and following premedication with 3 mg/kg atropine sulfate and 5 mg/kg diazepam. Under sterile conditions, a miniature electrical stimulator was inserted subcutaneously over the *vastus lateralis* muscle; electrodes were tunneled subcutaneously and sutured close to the common *peroneal* nerve. The incisions were closed in layers and the animals returned to individual cages with access to food and water *ad libitum*. Each stimulator was programed to deliver 0.2 ms rectangular wave pulses at 10 Hz. Electrical stimulation was initiated at the time of implantation. Pulsating contractions of the anterior compartment muscles innervated by the common *peroneal* nerve were palpable, producing oscillations of the foot when the leg was suspended, but the animals undergoing stimulation exhibited no signs of distress; they gained weight normally, and showed normal eating, sleeping, and locomotor behavior. Both hyper- and hypothyroid rabbits were exposed to 10 days of electrical stimulation to their left *peroneal* nerve. CMNS failed in one hypothyroid rabbit and three hyperthyroid rabbits and these rabbits were excluded from subsequent analysis. Euthyroid control rabbits (*n* = 2) received 21 days of CMNS before tissue harvesting.

Rabbits were anesthetized with 0.5 ml/kg of a 25% solution of urethane administered intravenously, followed by sodium pentobarbital until withdrawal reflexes were abolished. The *extensor digitorum longus* muscles from the stimulated limb, and the control muscles from the contralateral unstimulated limb, were dissected and rinsed briefly in sterile saline at 0–4°C, blotted onto sterile gauze, frozen in liquid nitrogen, and stored at −70°C. All animal experimental protocols were approved by and carried out in accordance with the recommendations of the Duke Institutional Animal Care and Use Committee (IACUC) of the Duke University School of Medicine.

### Western Blot

A total of 20–40 mg *extensor digitorum longus* muscles were homogenized in 400 μL of cold RIPA buffer (50 mM Tris, pH 8.0, 150 mM NaCl, 1% Triton X-100, 1% SDS, 0.5% sodium deoxycholate) supplemented with phosphatase inhibitor and protease inhibitor (Sigma), using MagNA Lyser (Roche). Protein concentration was determined by BCA assay kit (Thermo Scientific). Then, 40 μg of protein was separated by SDS-PAGE on 6-16% gradient Tris-glycine gels and transferred to 0.45 mM polyvinylidene fluoride membrane (Millipore). Membranes were blocked in 5% milk/PBST, and antibodies were incubated overnight at 4°C in 1% BSA/PBST. The membranes were incubated with antibodies to MHC-1, MHC-2a, LC3b-II, p62, p-70S6K, total p70S6K, p-AMPK, AMPK, p-ATGL, ATGL, p-AKT, AKT, GLUT4, CPT-1α, and skeletal muscle β-actin. This was followed by incubating with the secondary antibody conjugated with horseradish peroxidase and visualized using the enhanced chemiluminescence system (GE Healthcare). Densitometric analyses of western blot images were performed by using Image J software (NIH, Bethesda, MD, United States). Phosphorylated protein was normalized to total protein, and others were normalized to alpha-skeletal actin. We used the following antibodies to detect the target proteins: Cell Signaling Technology (Danvers, MA): LC3B (2775), p62 (5114), AMPK (5831), p-AMPK (Thr172) (2535), p-P70S6K (Thr389) (9205), P70S6K (9202), AKT (9272), and p-AKT (4060); Developmental Studies Hybridoma Bnak (University of Iowa, Iowa City, Iowa): MHCI (BA-D5), MHC2A (SC-71); Santa Cruz Biotechnology (Dallas, TX, United States): actin (sc-47778), Abcam (Cambridge, United Kingdom): p-ATGL (Ser406) (ab135093), ATGL (ab99532), CPT1α (ab128568), GLUT4 (ab654), GLUT1 (ab652), ATP5A and UQCR2 were detected using Total OXPHOS Rodent WB Antibody Cocktail (ab110413), and TFEB (ab2636); Millipore (Darmstadt, Germany): p-TFEB (Ser142) (ABE1971).

### Metabolomics

In accordance with previously established mass spectrometry (MS)-based methods, *extensor digitorum longus* muscle samples were used for quantitative determination of targeted metabolite levels for acylcarnitine species and organic acids by the Metabolomics Core Facility of Duke-NUS Medical School (Sinha et al., 2014; Farah et al., 2017). Muscle tissue was homogenized in 50% acetonitrile and 0.3% formic acid. For acylcarnitine and organic acid extraction, 100 μL of tissue homogenate was extracted using methanol. The acylcarnitine extracts were derivatized with 3 M hydrochloric acid in methanol, dried, and reconstituted in methanol for analysis in liquid chromatography/mass spectrometry (LC/MS). Acylcarnitine measurements were made using flow injection–tandem mass spectrometry on the Agilent 6430 Triple Quadrupole LC/MS system (Agilent Technologies, CA, United States). The sample analysis was carried out at 0.4 ml/min of 80:20 methanol:water as mobile phase and injection of 2 μL of sample. Data acquisition and analysis were performed on Agilent MassHunter Workstation B.06.00 software. Organic acid extracts were derivatized to form butyl esters using 3 M HCl in butanol. Samples were then reconstituted in 80% aqueous methanol and 4 μL of this solution was injected into an Agilent SB-C8 column (12 × 50 mm with 1.8 μm particle size) for analysis. Mobile phase was 80% methanol and 20% water, and flow rate was maintained at 0.4 mL/min for 2 min. Isocratic flow of 0.6 ml/min of 30% acetonitrile and 70% water with 0.1% formic acid was maintained for 5.5 min.

### Statistical Analysis

In Western blots, intensity of signals were assessed with densitometry; values for stimulated versus unstimulated muscles in the hypo- or hyperthyroid states were compared with Student’s *t*-test for paired variables. Signals in unstimulated muscles in eu-, hyper-, and hyperthyroid states were assessed by parametric one way ANOVA followed by Fisher’s least significant difference (LSD) multiple comparison test. Metabolomics and mRNA expression assessments were analyzed by two way repeated measures ANOVA followed by Fisher’s LSD multiple comparisons test. Total and free T3, heart rate, and change in body weight were analyzed using Student’s *t*-test. Statistical significance was declared at a *p*-value of <0.05. All statistical analyses were conducted in GraphPad Prism and R.

## Results

### CMNS Increased MHC-I Protein Expression in Hypothyroid Rabbits, but Not in Hyperthyroid Rabbits

**FIGURE 2:**
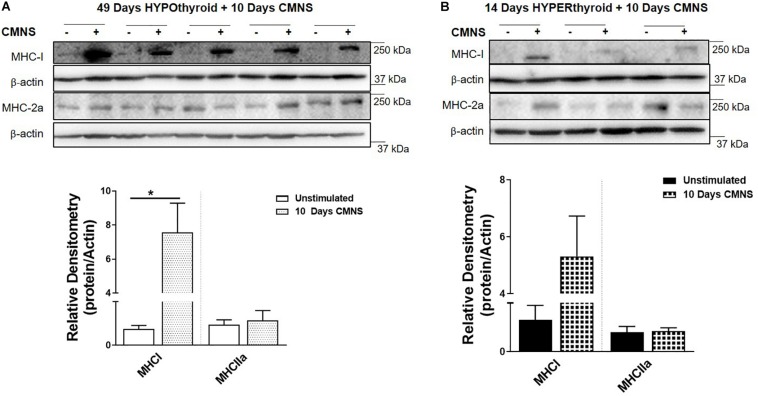
The impact of 10 days CMNS on protein levels of MHC-I and MHC-2a in hypothyroid and hyperthyroid rabbits. **(A,B)** Immunoblot and densitometric analysis of MHC-I and MHC-2a in the EDL muscle with or without CMNS from hypothyroid **(A)** (*n* = 5) or hyperthyroid **(B)** (*n* = 3) rabbits. Reassembled non-contiguous gel lanes are designated by black boxes. Densitometric values for stimulated versus unstimulated muscles in the hypo- or hyperthyroid states were compared with Student’s *t*-test for paired variables. Data are shown as mean ± SEM. ^∗^*P* < 0.01.

In order to confirm effective manipulation of thyroid status, we measured serum T_3_ levels, heart rate, and body weight (Supplementary Figures 1–3). After 10 days of CMNS, we observed that CMNS increased MHC-I protein expression in EDL muscle of hypothyroid rabbits (*p* < 0.01; Figure 2A). A similar effect was also seen in skeletal muscle of euthyroid rabbits after 21 days CMNS (Supplementary Figure 4). In contrast, 10 days of CMNS did not increase MHC-I in hyperthyroid rabbits (Figure 2B). Our group previously observed that T_3_ increased conversion of MHC-I to MHC-2a in the *soleus* muscle of mice (Lesmana et al., 2016); however, we did not observe this effect in EDL muscle of hyperthyroid rabbits as MHC-I was expressed at low levels in both unstimulated and CMNS-stimulated skeletal muscle (Figure 2B).

### CMNS Enhanced AKT-mTOR Signaling, and Expression of Glucose Transporter GLUT4 in Hypothyroid Rabbits

**FIGURE 3:**
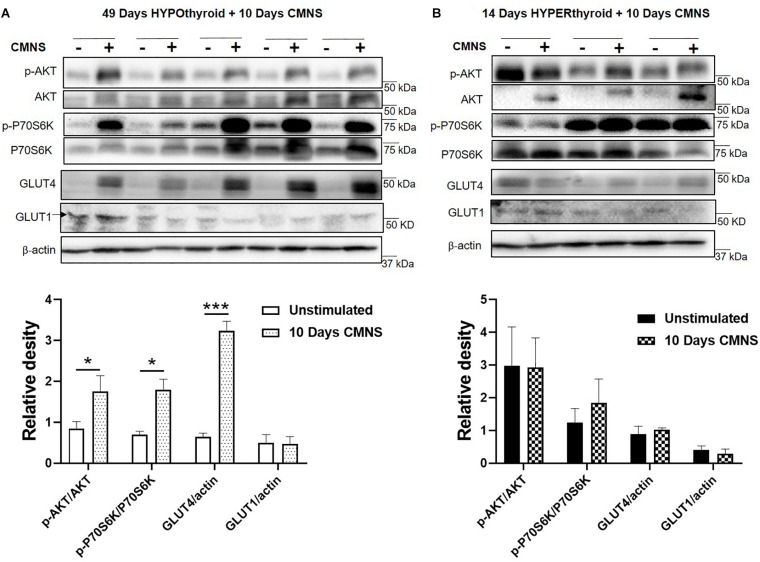
The impact of 10 days CMNS on AKT-mTOR signaling in hypothyroid and hyperthyroid rabbits. **(A,B)** Immunoblot and densitometric analysis of p-AKT/AKT, p-P70S6K/P70S6K, GLUT4, and GLUT1 in the EDL muscle with or without CMNS from hypothyroid **(A)** (*n* = 5) or hyperthyroid **(B)** (*n* = 3) rabbits. Reassembled non-contiguous gel lanes are designated by black boxes. Densitometric values for stimulated versus unstimulated muscles in the hypo- or hyperthyroid states were compared with Student’s *t*-test for paired variables. Data are shown as mean ± SEM. ^∗^*P* < 0.05, ^∗∗∗^*P* < 0.001.

In hypothyroid rabbits, 10 days of CMNS increased activation of AKT (*p* < 0.05), and P70S6K (*p* < 0.05) in the EDL muscle, indicating increased AKT-mTOR signaling in hypothyroid rabbits. Tissue protein concentration of GLUT4 was also increased by CMNS (*p* < 0.05), while GLUT1 remained unchanged (Figure 3A). In hyperthyroid rabbits, since they already were increased by hyperthyroidism (Supplementary Figure 5), CMNS did not affect the phosphorylation of AKT or P70S6K (Figure 3B).

### CMNS Enhanced AMPK Signaling and Expression of Mitochondrial Enzymes in Hypothyroid Rabbits

**FIGURE 4:**
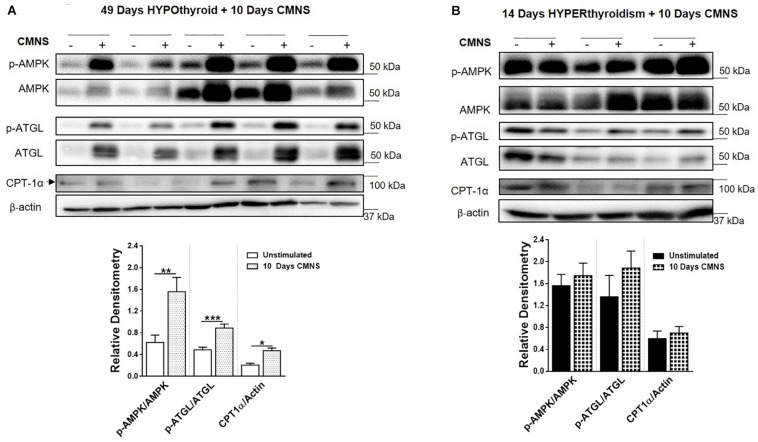
The impact of 10 days CMNS on AMPK signaling in hypothyroid and hyperthyroid rabbits. **(A,B)** Immunoblot and densitometric analysis of p-AMPK/AMPK, p-ATGL/ATGL, CPT-1α in the EDL muscle with or without CMNS from hypothyroid **(A)** (*n* = 5) or hyperthyroid **(B)** (*n* = 3) rabbits. Reassembled non-contiguous gel lanes are designated by black boxes. Densitometric values for stimulated versus unstimulated muscles in the hypo- or hyperthyroid states were compared with Student’s *t*-test for paired variables. Data are shown as mean ± SEM, ^∗^*P* < 0.05, ^∗∗^*P* < 0.01, ^∗∗∗^*P* < 0.001.

**FIGURE 5:**
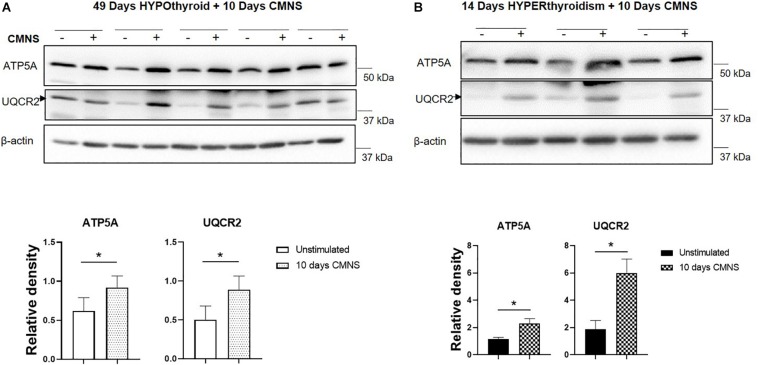
The impact of 10 days CMNS on ATP5A and UQCR2 in hypothyroid and hyperthyroid rabbits. **(A,B)** Immunoblot and densitometric analysis of ATP5A, UQCR2, in the EDL muscle with or without CMNS from hypothyroid **(A)** (*n* = 5) or hyperthyroid **(B)** (*n* = 3) rabbits. Reassembled non-contiguous gel lanes are designated by black boxes. Densitometric values for stimulated versus unstimulated muscles in the hypo- or hyperthyroid states were compared with Student’s *t*-test for paired variables. Data are shown as mean ± SEM, ^∗^*P* < 0.05.

Exercise generates an increased energy demand in skeletal muscle, which is tightly coupled with AMPK activation (Chen et al., 2003). In hypothyroid rabbits, 10 days of CMNS increased phosphorylation of AMPK (*p* < 0.01; Figure 4A). Furthermore, 10 days of CMNS also increased the phosphorylation of ATGL at Ser^406^ (*p* < 0.001, Figure 4A), a site phosphorylated by AMPK to enhance hydrolysis of triglycerides and promote fatty acid oxidation (Kim et al., 2016). CMNS also increased expression of CPT-1α (*p* < 0.05; Figure 4A), the protein facilitating transport of fatty acids into mitochondria. However, in hyperthyroid rabbits, phosphorylation of AMPK was already increased in the unstimulated skeletal muscle of hyperthyroid rabbits (Supplementary Figure 6), so CMNS did not cause any further increase (Figure 4B). Similarly, CMNS did not change *p*-ATGL or CPT-1α expression in skeletal muscle of hyperthyroid rabbits (Figure 4B). We further assessed the expression of several enzymes belonging to mitochondrial electron transport chain (ETC) complexes. We found that CMNS increased protein levels of ATP5A (ATP synthase F1 subunit alpha) (*p* < 0.05) and UQCR2 (*p* < 0.05), a subunit of the respiratory chain protein ubiquinol cytochrome c reductase, key components of mitochondrial complexes V and III, respectively (Figure 5). Interestingly, the increase of ATP5A and UQCR2 expression was observed in both hypo- and hyperthyroid rabbits, suggesting that mitochondrial ETC was increased by CMNS in both hypo- and hyperthyroidism (Figure 5).

### CMNS Enhanced Fatty Acid β-Oxidation and TCA Cycle Flux in Hypothyroid Rabbits

**FIGURE 6:**
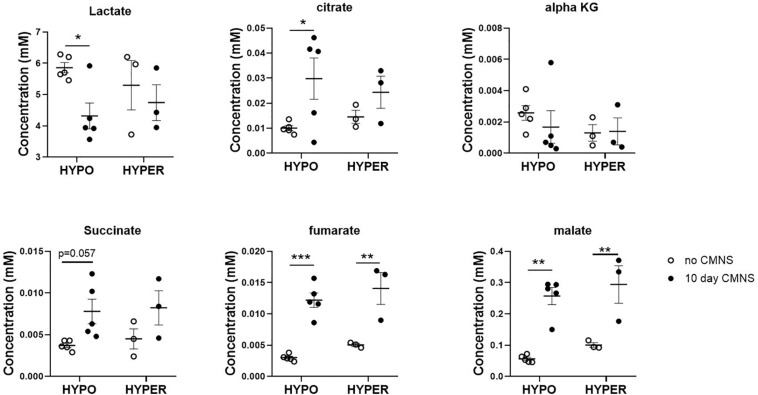
The levels of organic acids in the EDL muscle with or without 10 days CMNS from hypothyroid (*n* = 5) and hyperthyroid (*n* = 3) rabbits. Data were analyzed by two way repeated measures ANOVA followed by Fisher’s least significant difference multiple comparisons test. Data are shown as mean ± SEM, ^∗^*P* < 0.05, ^∗∗^*P* < 0.01, ^∗∗∗^*P* < 0.001.

**FIGURE 7:**
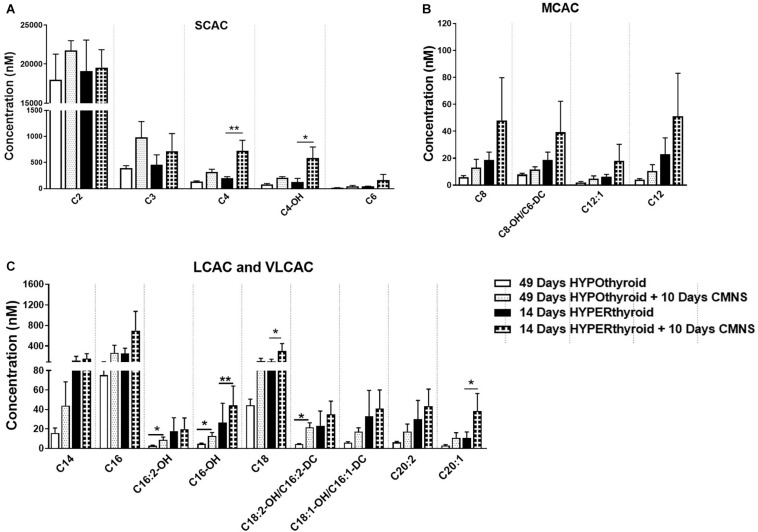
The levels of acylcarnitines in the EDL muscle with or without CMNS from hypothyroid (*n* = 5) and hyperthyroid (*n* = 3) rabbits. **(A)** Short chain acylcarnitines (SCAC). **(B)** Medium chain acylcarnitines (MCAC). **(C)** Long chain (LCAC) and very long chain acylcarnitines (VLCAC). Data were analyzed by two way repeated measures ANOVA followed by Fisher’s least significant difference multiple comparisons test. Data are shown as mean ± SEM, ^∗^*P* < 0.05, ^∗∗^*P* < 0.01.

It has been reported that thyroid hormone stimulates glycolysis and lipid oxidation through activation of AKT and AMPK respectively (de Lange et al., 2008). However, little is known about the impact of thyroid status on changes in glucose and lipid metabolism in response to CMNS. We next determined muscle content of small molecular intermediates of acylcarnitine and TCA metabolism in unstimulated and stimulated skeletal muscle from hypo- and hyperthyroid rabbits. Two-way ANOVA analysis indicated CMNS caused changes in several metabolites involved in TCA cycle and β-oxidation (Supplementary Tables 1–4). Ten days of CMNS decreased lactate, but increased the TCA cycle intermediates citrate, succinate, fumarate, and malate (Figure 6), suggesting that CMNS may increase aerobic glycolysis in the hyperthyroid rabbits due to increased AKT activity and GLUT4 expression. In hypothyroid rabbits, 10 days of CMNS increased skeletal muscle concentrations of short- and long-chain acylcarnitine species (Figure 7 and Supplementary Figures 7–9), suggesting that there was increased acylcarnitine flux due to increased β-oxidation of fatty acids.

### Effect of CMNS on Autophagic Markers

**FIGURE 8:**
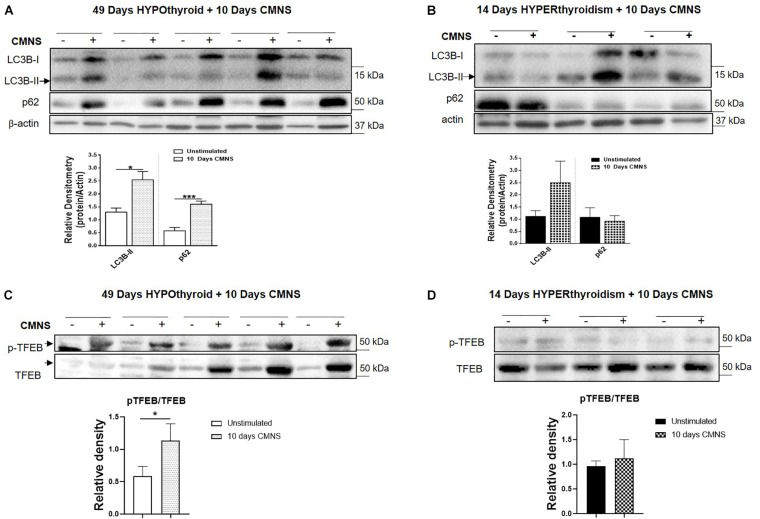
The impact of 10 days CMNS on protein levels of LC3B-II, p62, and p-TFEB in hypothyroid and hyperthyroid rabbits. **(A,B)** Immunoblot and densitometric analysis of LC3B-II and p62 in EDL muscle with or without CMNS from hypothyroid **(A)** (*n* = 5) or hyperthyroid **(B)** (*n* = 3) rabbits. **(C,D)** Immunoblot and densitometric analysis of p-TFEB/TFEB in EDL muscle with or without CMNS from hypothyroid **(C)** (*n* = 5) or hyperthyroid **(D)** (*n* = 3) rabbits. Reassembled non-contiguous gel lanes are designated by black boxes. Densitometric values for stimulated versus unstimulated muscles in the hypo- or hyperthyroid states were compared with Student’s *t*-test for paired variables. Data are shown as mean ± SEM, ^∗^*P* < 0.05, ^∗∗∗^*P* < 0.001.

In hypothyroid rabbits, 10 days of CMNS increased protein levels of LC3B-II (*p* < 0.05) and p62 (*p* < 0.01) proteins (Figure 8A), but decreased p62 mRNA expression (*p* < 0.05; Supplementary Figure 10B), suggesting that the accumulation of LC3B-II and p62 proteins may be due to an impairment of lysosomal degradation. We also assessed phosphorylation of TFEB, an important transcription factor for lysosomal biogenesis (Settembre et al., 2011). TFEB can be phosphorylated by mTOR, and its phosphorylated form is excluded from the nucleus resulting in decreased transcriptional activity (Martina et al., 2012). CMNS increased the phosphorylation of TFEB in hypothyroid rabbits (*p* < 0.05; Figure 8C). These results suggested that there could be impairment of autophagy in CMNS in hypothyroid rabbits. In hyperthyroid rabbits, 10 days of CMNS tended to increase skeletal muscle LC3B-II levels but these changes were not statistically significant. There was no change in p62 expression observed after 10 days of CMNS in hyperthyroid rabbits (Figure 8B). There were no changes in muscle mRNA concentrations of LC3B and p62 (Supplementary Figure 10) or phosphorylation of TFEB (Figure 8D). These findings suggest there were little or no autophagic responses to CMNS in the chronic hyperthyroid state.

## Discussion

In this manuscript, we examined the impact of two extremes of thyroid hormone status, hypo- and hyperthyroidism, on the structural and metabolic adaptations of skeletal muscle to exercise training in rabbits. For these studies, we used CMNS – a well-established animal model that simulates exercise training. While CMNS permits investigation of a chronic and exaggerated physiologic response to exercise training, CMNS differs from normal exercise training in that the stimulus is continuous; thus, CMNS generates a greater metabolic demand without an opportunity for recovery between exposure bouts. In order to evaluate the effects of manipulating TH status on CMNS responses while avoiding potentially confounding effects of variation among individual animals, we compared the stimulated and unstimulated muscles from opposite limbs of the same animals. We also employed rabbits since the skeletal muscle composition and the physiological response to exercise in larger mammals such as rabbits and primates more closely resemble those found in humans. For example, similar to humans, β-myosin heavy chain is the predominant sarcomeric myosin protein expressed in the cardiac ventricles of rabbits, whereas α-myosin heavy chain is the predominant sarcomeric myosin protein expressed in rodent cardiac ventricles (Cummins and Russell, 1986).

Using this well characterized *in vivo* model of exercise training, we observed that CMNS increased MHC-I expression in the skeletal muscle of hypothyroid rabbits consistent with the well-established increase in MHC-I fiber expression and overall muscle mass seen with chronic exercise training in smaller mammals (Booth and Thomason, 1991). In the hypothyroid state, CMNS induction of MHC-I could be due to increased protein synthesis from enhanced Akt and mTOR signaling and/or decreased degradation, perhaps due to suppression of autophagic pathways. We previously observed a decrease in MHC-I protein expression in *soleus* muscle when autophagy was stimulated by T_3_, and an increase when autophagy was blocked (Lesmana et al., 2016). Thus, the changes in LC3B-II and p62 suggest there could be decreased autophagy after exercise in hypothyroid rabbits. Several signaling pathways are involved in autophagy regulation, in particular, activation of AMPK signaling increases autophagy while mTOR signaling inhibits it (He and Klionsky, 2009; Kim et al., 2011). AMPK can inhibit mTOR, and both AMPK and mTOR phosphorylate ULK1 at different sites and to stimulate and inhibit autophagy, respectively (He and Klionsky, 2009; Kim et al., 2011; Hindupur et al., 2015). Interestingly, we observed the simultaneous activation of both AMPK and AKT-mTOR signaling by CMNS in EDL muscle of hypothyroid rabbits. In hypothyroid rabbits undergoing CMNS, the inhibitory effect of mTOR phosphorylation on autophagy appears to be dominant over the activating effect of AMPK phosphorylation since autophagy was decreased. Although the detailed mechanism underlying the dual activation of mTOR and AMPK in hypothyroid rabbits during CMNS is not known, it is noteworthy that concurrent activation of both pathways can be induced by amino acids in cultured myoblasts (Dalle Pezze et al., 2016). mTOR also can phosphorylate TFEB, a transcription factor that stimulates autophagy and lysosomal biogenesis, resulting in decreased nuclear localization of TFEB (Roczniak-Ferguson et al., 2012; Napolitano et al., 2018). We observed increased phosphorylation of TFEB at Ser142, the mTOR phosphorylation site (Roczniak-Ferguson et al., 2012), suggesting that CMNS could regulate autophagy through the mTOR/TFEB axis.

Increased muscle fiber content is associated with increased utilization of fatty acids as fuel; so, increased MHC-I would be expected to be associated with increased β-oxidation of fatty acids due to chronic exercise. Interestingly, in hypothyroid rabbits, we found that CMNS led to concomitant increases in fatty acid β-oxidation and glucose transporter expression; therefore, it is likely that both fuels were utilized in skeletal muscle adaptations to CMNS in the hypothyroid state. Moreover, in the EDL of hypothyroid rabbits, we observed that the lipase ATGL was activated by phosphorylation; whereas no induction of autophagy by CMNS was observed. We previously showed that lipophagy was an important mechanism for transporting lipid from fat droplets to lysosomes, and was critical for thyroid hormone-mediated autophagy and β-oxidation in the liver (Sinha et al., 2012). These findings suggest that during CMNS in the hypothyroid state, ATGL, rather than autophagy of lipids (lipophagy), supplied free fatty acids to mitochondria. It previously was reported that acute bouts of exercise increase skeletal muscle autophagy (Sanchez et al., 2014); however, our results suggest that autophagy may be impaired (in hypothyroid rabbits) or not affected (in hyperthyroid rabbits) by CMNS. Thus, it is possible that autophagy may be up-regulated in response to acute exercise; but, this response may diminish during chronic exercise stimulated by CMNS, particularly during altered TH states. It also is interesting to speculate that lipophagy may provide fatty acids for mitochondrial β-oxidation in untrained muscle, whereas ATGL may predominantly generate fatty acids for mitochondria in trained muscle.

In the hypothyroid rabbits, we observed concomitant induction of mTOR and AMPK with CMNS. The phosphorylation of AMPK blocks fatty acid synthesis by virtue of its phosphorylation and inactivation of acetyl-CoA carboxylase (ACC). This leads to decreased malonyl-CoA, an inhibitor of the fatty acid carrier protein, CPT-1α, and results in increased delivery of fatty acids into the mitochondria for β-oxidation. Indeed, in hypothyroid rabbits, we observed that CMNS increased the concentration of intermediates of acylcarnitine metabolism. We also observed increased Akt-mTOR signaling, which correlated with increased protein levels of GLUT4, aerobic glycolysis, and inhibits autophagy. In hypothyroid rabbits, CMNS increased TCA metabolites, suggesting enhanced TCA flux due to increased fatty acid metabolism and oxidative phosphorylation. Interestingly, exercise increases GLUT4 protein level in skeletal muscle (Gurley et al., 2016). Our observation of an increase in p-AKT and GLUT4 expression and the concomitant decrease in lactate levels supports the case for an increase in aerobic glycolysis. Indeed, it is remarkable that in hypothyroid rabbits, CMNS increased β-oxidation of fatty acids despite activation of Akt and enhanced glucose transport/aerobic glycolysis. In addition to increasing glucose metabolism, activation of Akt and mTOR also increases protein synthesis (Bodine et al., 2001); thus, this may represent one mechanism by which exercise training increases AMPK phosphorylation to promote fatty acid β-oxidation, while at the same time increasing Akt phosphorylation to stimulate protein synthesis and muscle hypertrophy.

In hyperthyroid rabbits, MHC-1 protein content was low. CMNS did not increase MHC-I protein or cause a shift in fiber type from MHC-I to MHC-2. Additionally, mTOR-mediated protein synthesis was activated at baseline and did not increase further in response to CMNS. In hyperthyroid rabbits, CMNS caused little or no increase in autophagy. Interestingly, the expression of ATP5A and UQCR2, the subunits of mitochondrial complex V and III, respectively, were increased in both hypo- and hyperthyroid rabbits by CMNS. These findings are consistent with the shift toward glycolysis observed in muscle by TH treatment as well as the tendency for CMNS to increase fatty acid β-oxidation. Indeed, we observed that even in the hyperthyroid rabbits, CMNS also increased the concentrations of several TCA metabolites and acylcarnitine species.

Our work has several strengths including the use of the well-characterized model of CMNS, the use of a larger mammal with a basal metabolic rate more similar to humans, and the extensive molecular evaluation of the responses to CMNS at the extremes of thyroid status. The major limitation is the paucity of data in euthyroid animals; however, by studying the response to CMNS at the extremes of thyroid status, this relative limitation is minimized.

**FIGURE 9:**
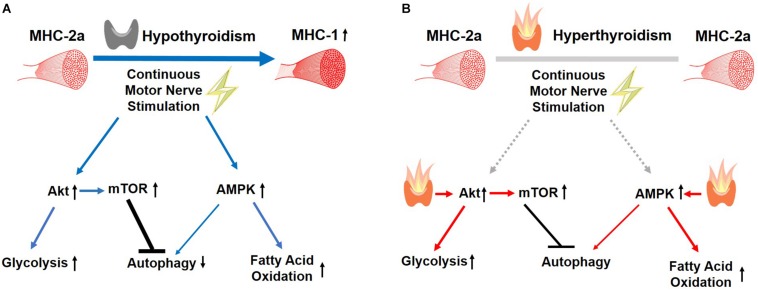
Summary of the effects of CMNS on skeletal muscle from hypothyroid and hyperthyroid rabbits. The stimulating, or inhibitory effects of CMNS are indicated by the blue arrows or black lines, respectively. In the hypothyroid state **(A)**, CMNS increases synthesis of MHC-I; AKT-mTOR signaling and glucose utilization; AMPK signaling and fatty acid oxidation; while decreasing autophagy. In the hyperthyroid state **(B)**, those effects were lost or less significant in hyperthyroid rabbits (indicated by gray lines), as those changes were already induced by thyroid hormone prior to CMNS stimulation (indicated by red arrows). Images used in Figure 9 adapted from https://www.kissclipart.com/muscle-clip-art-clipart-skeletal-muscle-clip-art-v7dy09/, https://commons.wikimedia.org/wiki/File:201405_thyroid_gland.png, and https://svgsilh.com/ff5722/image/2747390.html.

In summary, our findings support a permissive muscle response to CMNS during hypothyroidism that was not observed during hyperthyroidism (Figure 9). CMNS increased muscle fiber type MHC-I expression, and simultaneously increased AMPK and mTOR signaling in hypothyroid rabbits whereas it had little or no effect on these processes in hyperthyroid rabbits. In hypothyroid rabbits, CMNS increased phosphorylation of AMPK to enable the use of fatty acids and glucose as fuels, while it also increased phosphorylation of mTOR to stimulate the protein synthesis necessary for the growth of muscle mass that occurs during exercise training. Since thyroid status varies across mammalian species and inversely correlates with size, thyroid status should be considered when designing experiments and interpreting results of chronic exercise in animals and humans. Thus, it appears that thyroid hormone dominates cellular responses to exercise training in skeletal muscle and that assessment of thyroid hormone status may be important in assessing adaptation to training in humans.

## Data Availability Statement

The datasets generated for this study are available on request to the corresponding author.

## Ethics Statement

All animal experimental protocols were approved by and carried out in accordance with the recommendations of the Duke Institutional Animal Care and Use Committee (IACUC) of the Duke University School of Medicine.

## Author Contributions

JZ performed the assays described in the manuscript, contributed to the statistical analysis and draft revisions, participated in the interpretation of the results, and produced the initial draft of the manuscript and figures. DP contributed to the statistical analysis and draft revisions, participated in the interpretation of the results, and produced the initial draft of the manuscript and figures. AL and JH contributed to the data acquisition. WK conducted the original animal experiments under the auspices of the Duke University IUCAC, participated in the interpretation of the results, and contributed to the draft revisions. JW, KH, and PY participated in the interpretation of the results and contributed to the draft revisions.

## Conflict of Interest

The authors declare that the research was conducted in the absence of any commercial or financial relationships that could be construed as a potential conflict of interest.
