# Supplementary material for: Thyroid Hormone Status Regulates Skeletal Muscle Response to Chronic Motor Nerve Stimulation
Source: Front Physiol. 2019 Oct 31;10:1363. doi: 10.3389/fphys.2019.01363 (PMC6834779; doi:10.3389/fphys.2019.01363)
Supplement: Supplementary file 1 [file Table_1.docx]

**Supplementary Figure 1.** Serum total and free T_3_ concentrations in hypothyroid and hyperthyroid rabbits. Total and Free T3 concentrations were quantified in sera after CMNS. We were unable to obtain T_3_ and fT_3_ measurements for all animals included in the study. For the purposes of statistical comparison, the hyperthyroid condition includes data from animals in whom CMNS failed. Hyperthyroid rabbits had significantly higher concentrations of both total and free T_3_ compared to hypothyroid rabbits. Data were analyzed using Student’s t-test (*p<0.05).


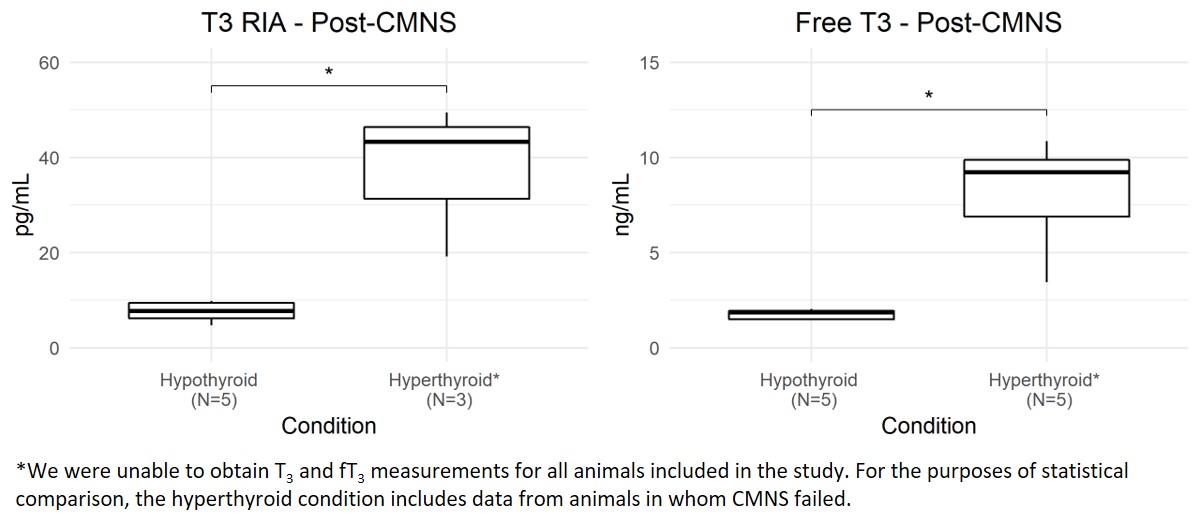


**Supplementary Figure 2.** Heart rate in hypothyroid and hyperthyroid rabbits**.** Heart rate was measured using a single lead electrocardiogram. Hyperthyroid (n=5) rabbits had a significantly greater heart rate compared to hypothyroid (n=3) rabbits. Data were analyzed using Student’s t-test (*p<0.05).


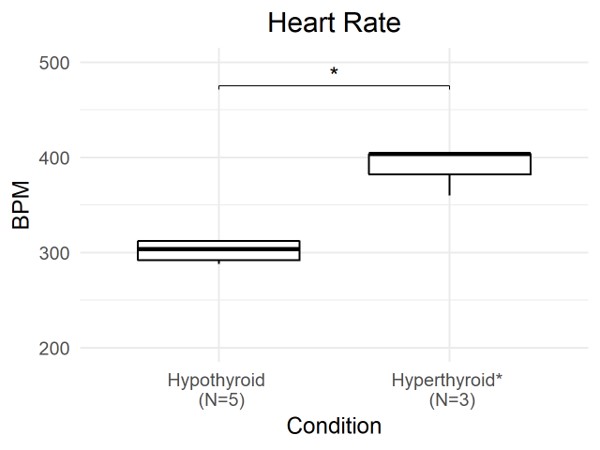


**Supplementary Figure 3.** Change in bodyweight with thyroid status manipulation. After manipulation of thyroid status, hypothyroid (n=5) rabbits experienced an increase in weight while hyperthyroid (n=3) rabbits remained weight stable. Data were analyzed using Student’s t-test (*p<0.05).


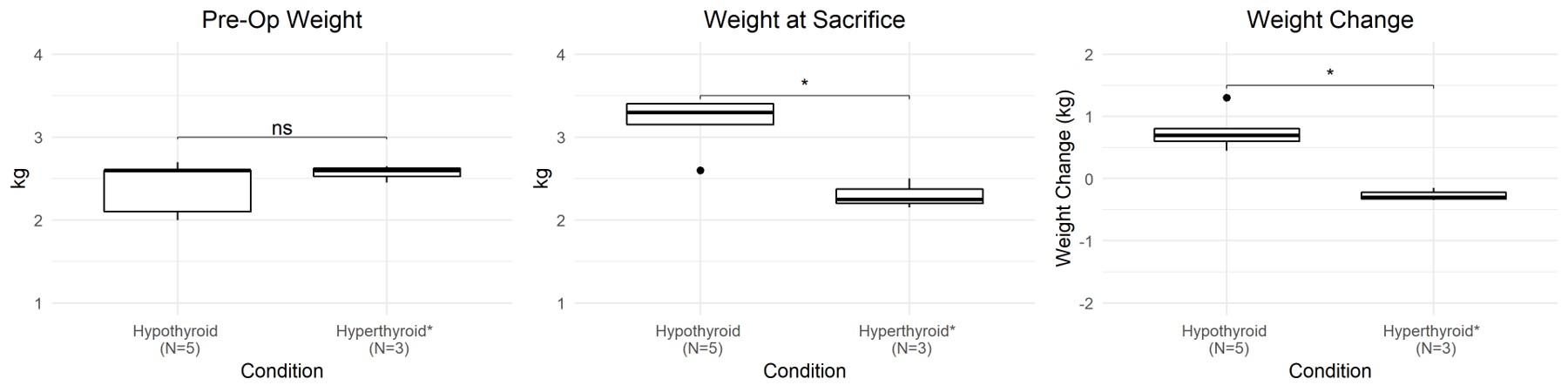


**Supplementary Figure 4.** The impact of 21 days CMNS on protein levels of MHC-I and MHC-2a in euthyroid rabbits.


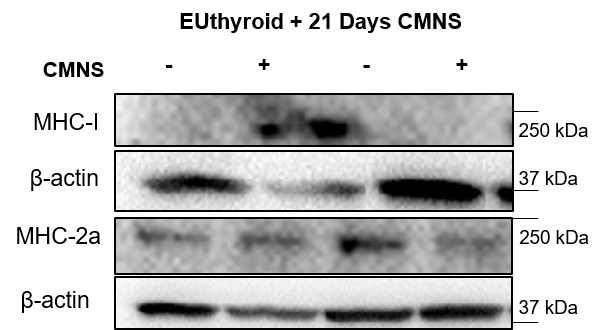


**Supplementary Figure 5.** The impact of thyroid status on the activation of AKT and P70S6K in the unstimulated EDL muscle from euthyroid (n=3), hyperthyroid (n=3) and hypothyroid (n=5) rabbits. Immunoblot and densitometric analysis of p-AKT/AKT, and p-P70S6K/P70S6K. Data were analyzed by parametric one way ANOVA followed by Fisher’s LSD multiple comparison test. Data are shown as mean ±SEM, *p<0.05.


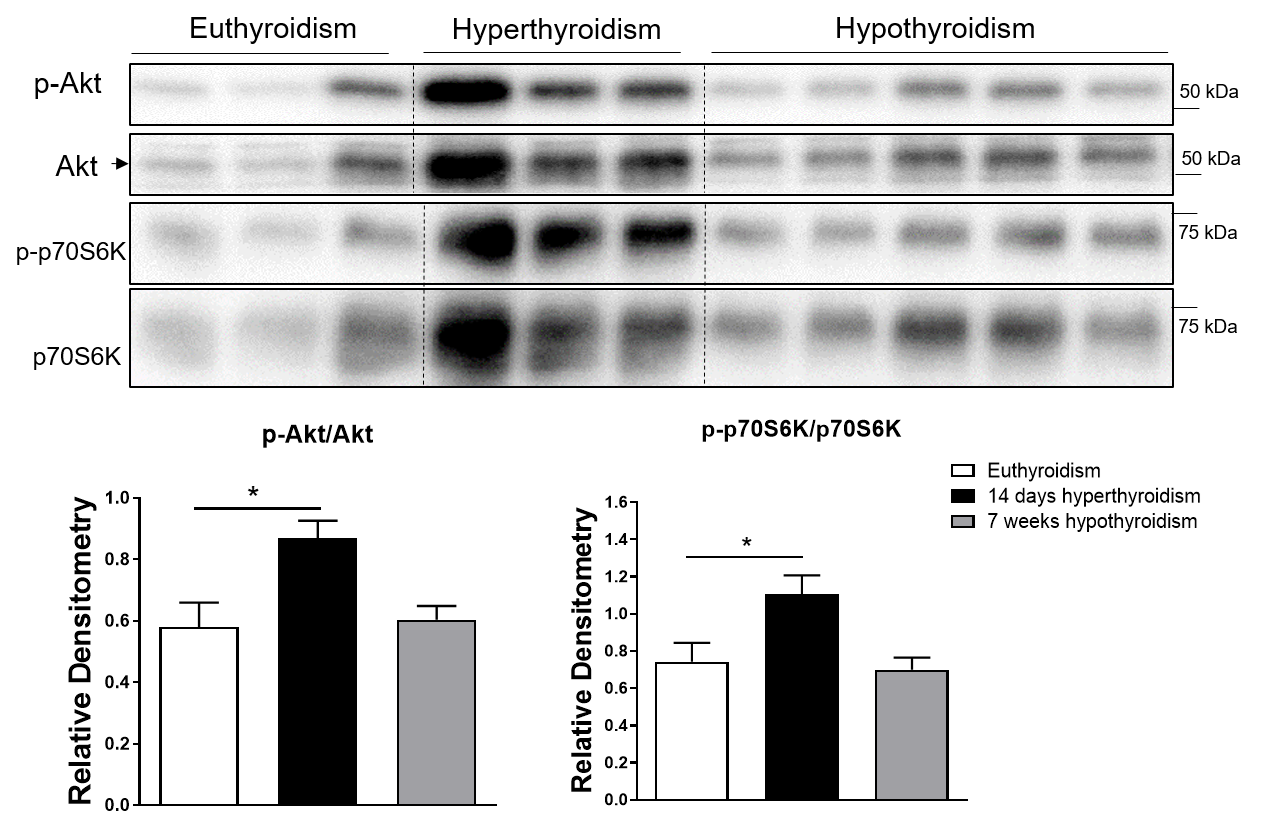


**Supplementary Figure 6.** The impact of thyroid status on the activation of AMPK in the unstimulated EDL muscle from euthyroid (n=3), hyperthyroid (n=3) and hypothyroid (n=5) rabbits. Immunoblot and densitometric analysis of p-AMPK/AMPK. Data were analyzed by parametric one way ANOVA followed by Fisher’s LSD multiple comparison test. Data are shown as mean ±SEM, **p<0.01.


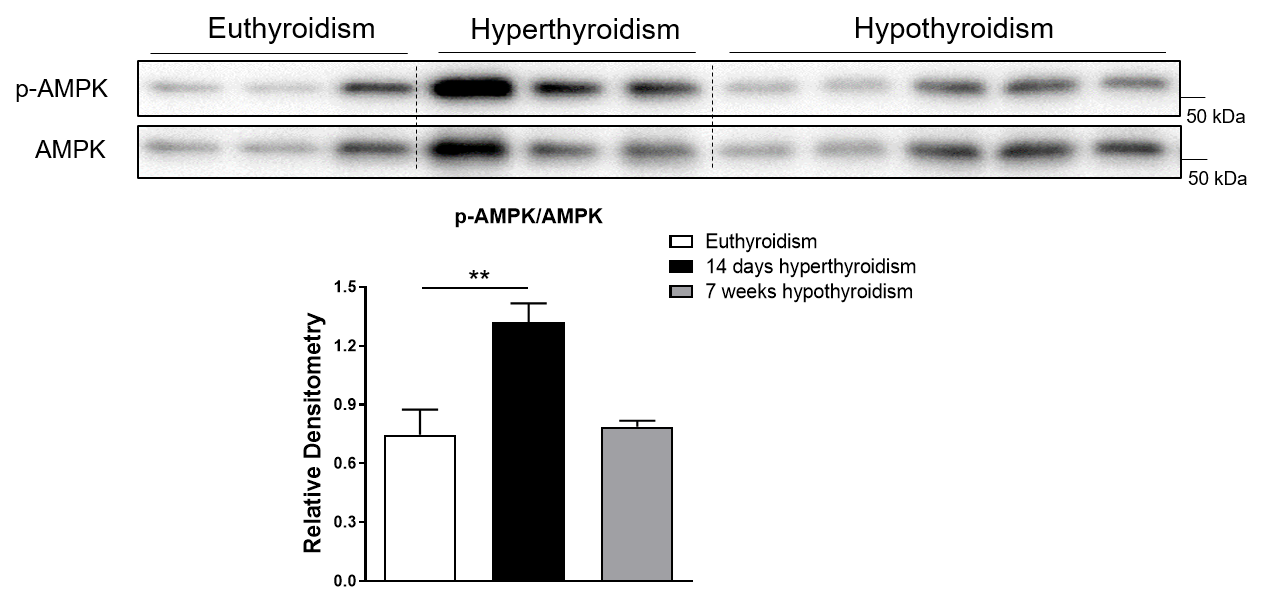


**Supplementary Figure 7.** Scatter plot showing the individual short chain acylcarnitine species in the stimulated or unstimulated EDL muscle from hypothyroid (n=5) or hyperthyroid (n=3) rabbit. Data were analyzed by two way repeated measures ANOVA followed by Fisher’s least significant difference multiple comparisons test. Data are shown as mean ±SEM, *p<0.05.


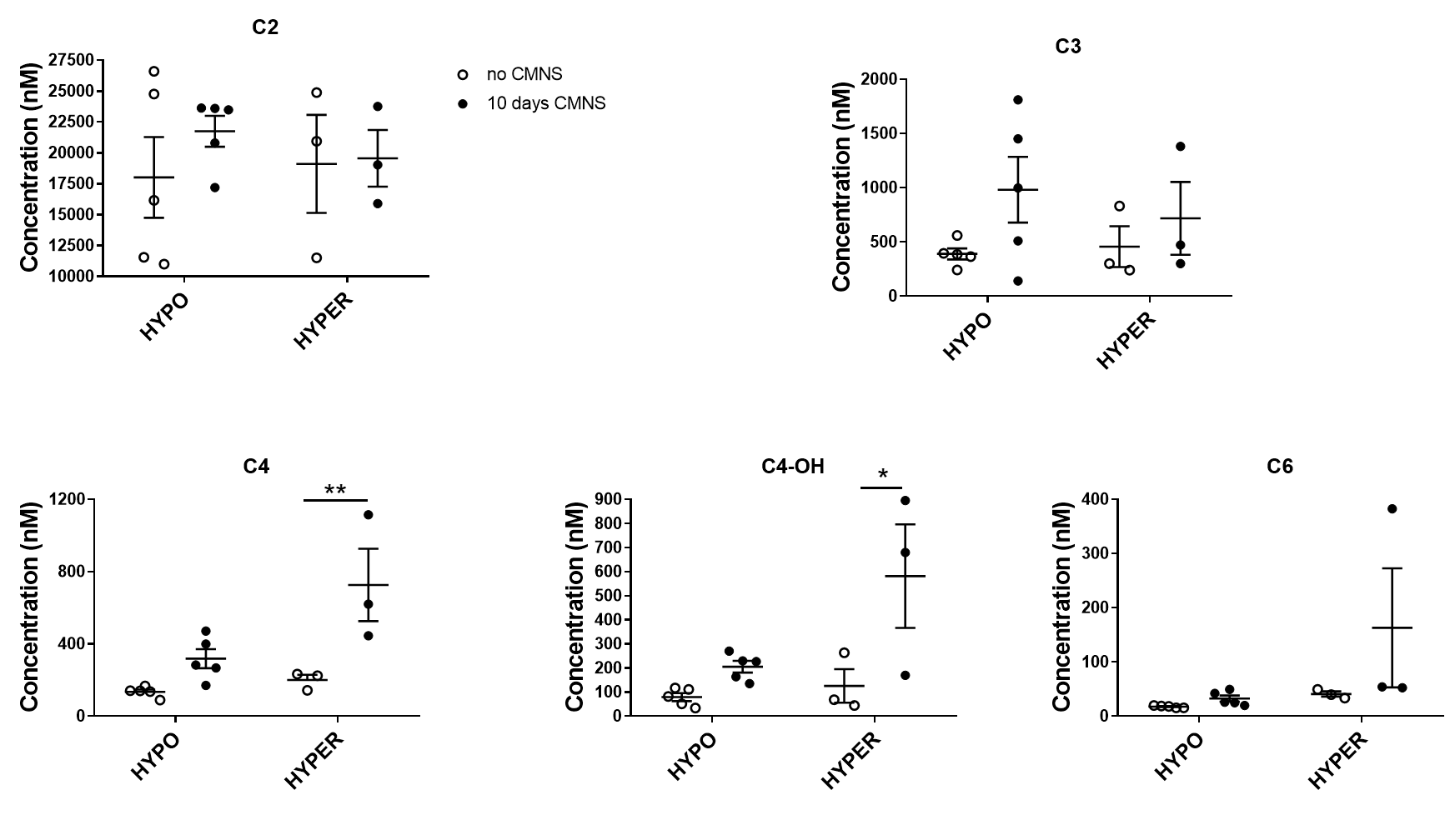


**Supplementary Figure 8.** Scatter plot showing the individual medium chain acylcarnitine species in the stimulated or unstimulated EDL muscle from hypothyroid (n=5) or hyperthyroid (n=3) rabbit. Data were analyzed by two way repeated measures ANOVA followed by Fisher’s least significant difference multiple comparisons test. Data are shown as mean ±SEM.


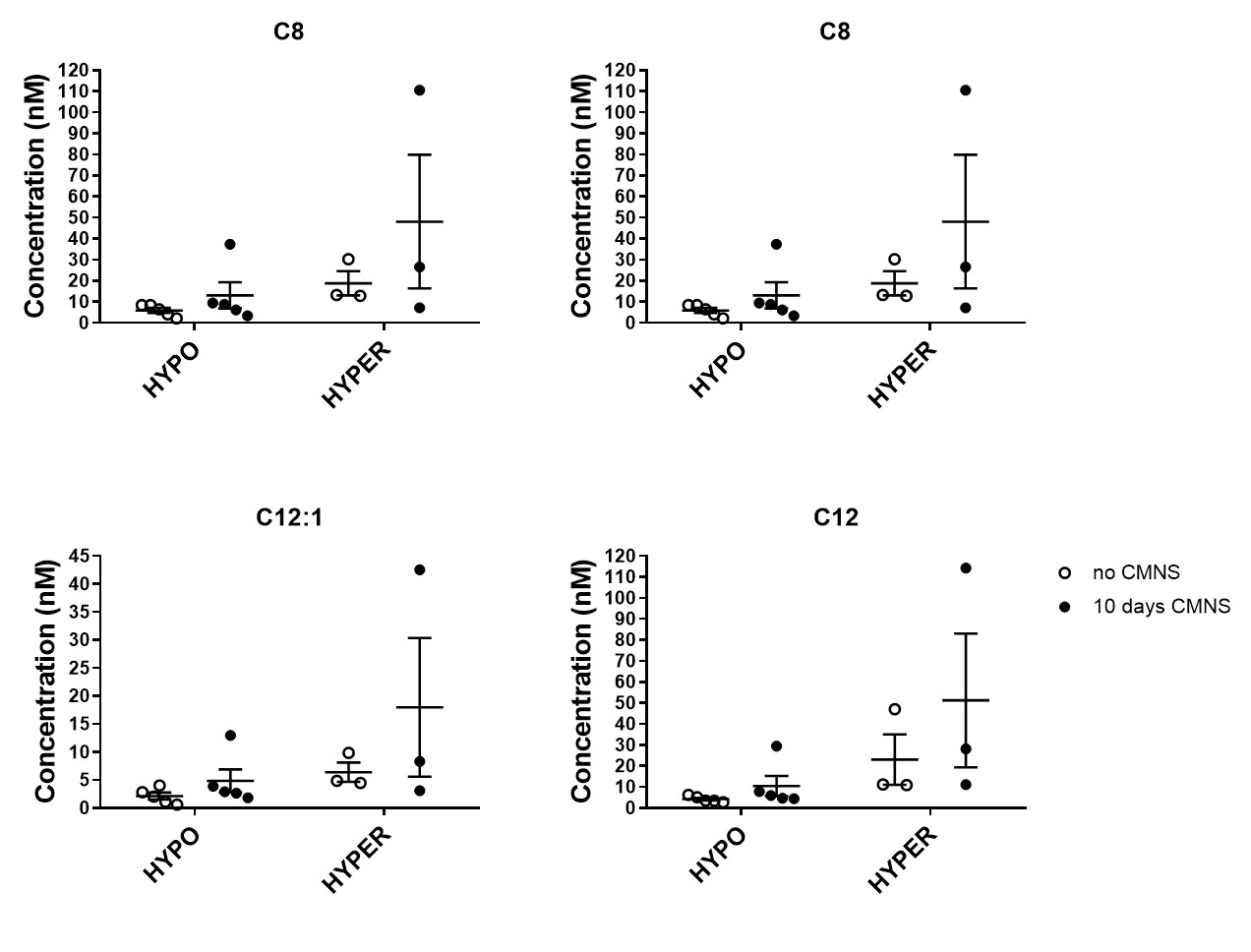


**Supplementary Figure 9.** Scatter plot showing the individual long chain and very long chain acylcarnitine species in the stimulated or unstimulated EDL muscle from hypothyroid (n=5) or hyperthyroid (n=3) rabbit. Data were analyzed by two way repeated measures ANOVA followed by Fisher’s least significant difference multiple comparisons test. Data are shown as mean ±SEM, *p<0.05, **p<0.01.


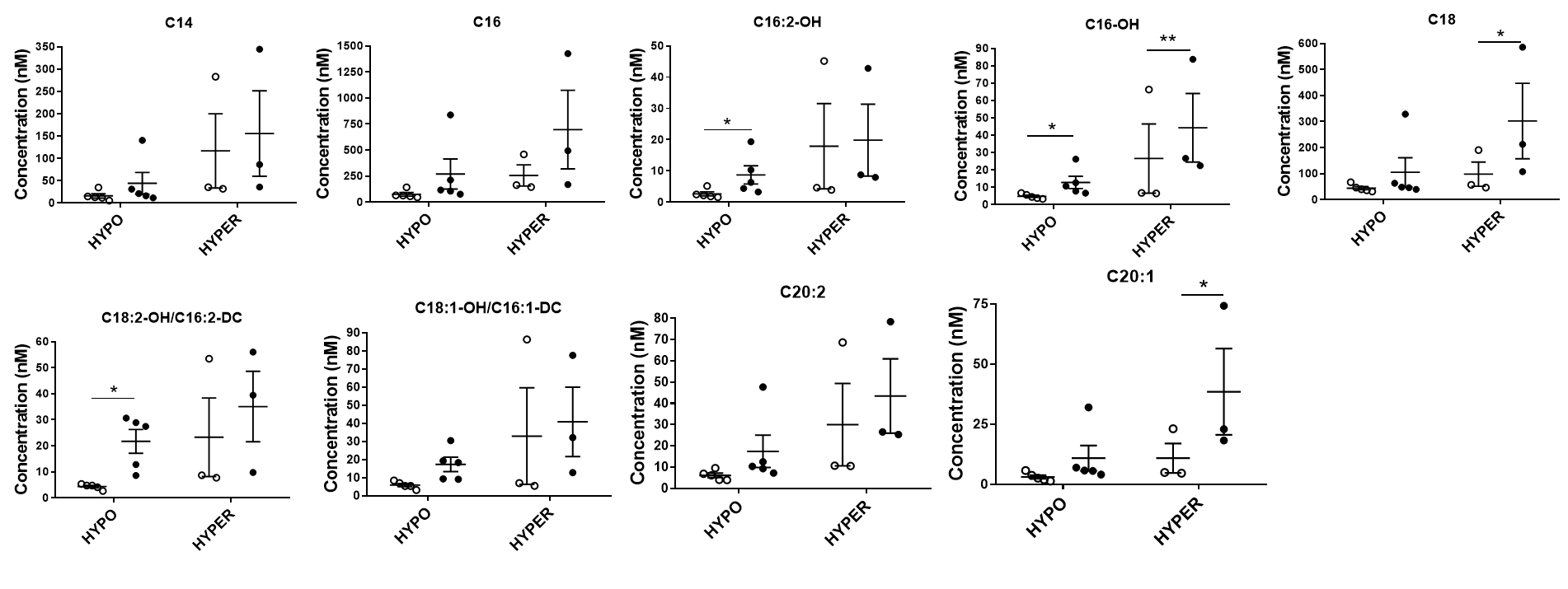


**Supplementary Figure 10.** The impact of 10 days CMNS on mRNA levels of LC3B (A), and p62 (B) in EDL muscle with or without CMNS from hypothyroid (n=5) or hyperthyroid (n=3) rabbits. We observed no significant difference in mRNA level of LC3B (two way repeated measures ANOVA thyroid status X CMNS P = 0.5318) or p62 (two way repeated measures ANOVA thyroid status X CMNS P = 0.1726). Data were analyzed by two way repeated measures ANOVA followed by Fisher’s least significant difference multiple comparisons test. Data are shown as mean ±SEM.


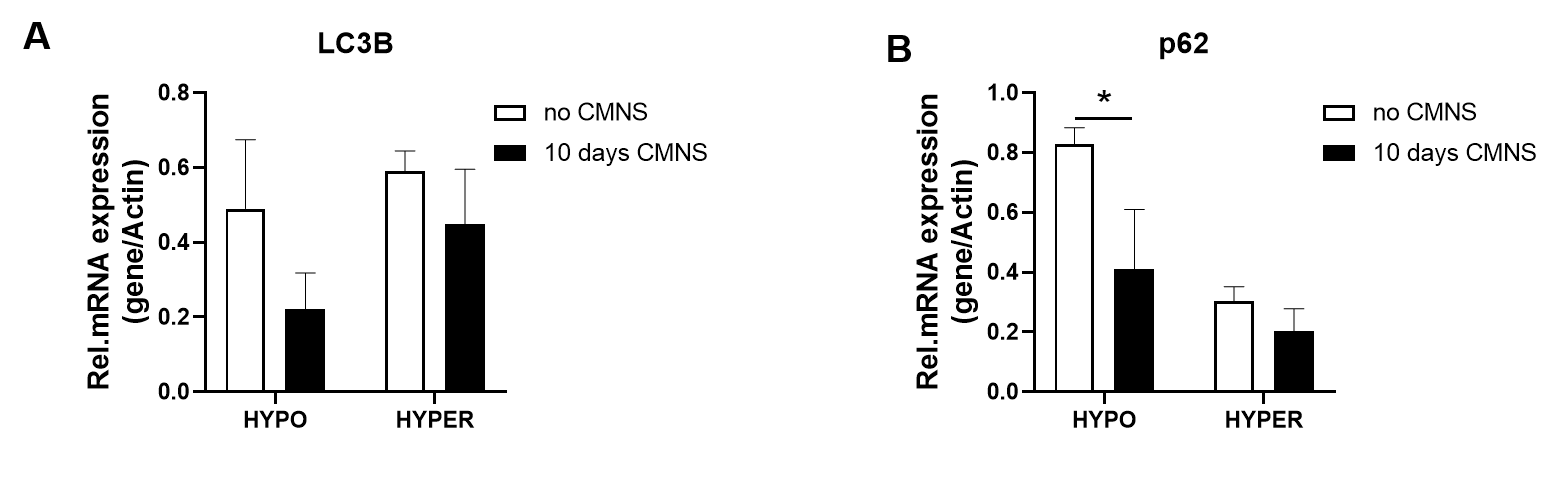


**Supplementary Table 1.** Two-way repeated measures ANOVA analysis of TCA cycle intermediates**.**

**
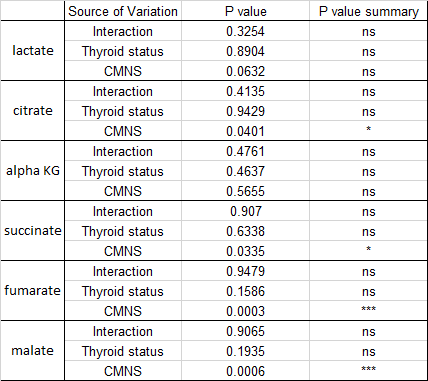
**

**Supplementary Table 2.** Two-way repeated measures ANOVA analysis of SCAC species**.**

**
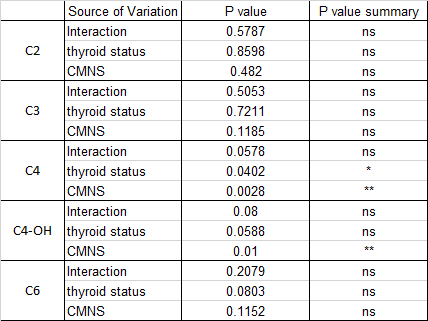
**

**Supplementary Table 3.** Two-way repeated measures ANOVA analysis of MCAC species**.**

**
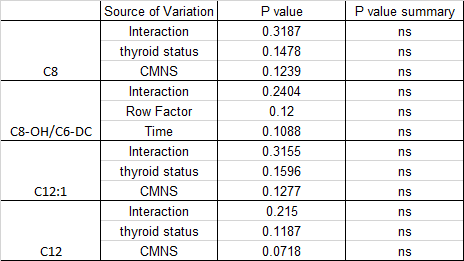
**

**Supplementary Table 4.** Two-way repeated measures ANOVA analysis of LCAC and VLCAC species**.**

**
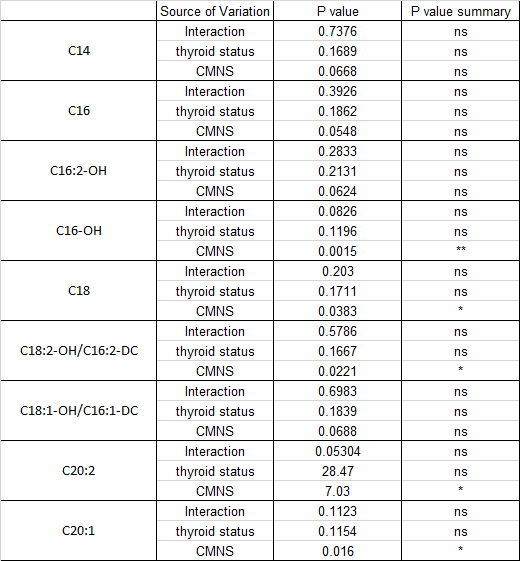
**
